# Nanomechanical Motion Transducers for Miniaturized Mechanical Systems

**DOI:** 10.3390/mi8040108

**Published:** 2017-04-01

**Authors:** Taejoon Kouh, M. Selim Hanay, Kamil L. Ekinci

**Affiliations:** 1 Department of Physics, Kookmin University, Seoul 136-702, Korea; 2 Department of Mechanical Engineering, and the National Nanotechnology Research Center (UNAM), Bilkent University, Ankara 06800, Turkey; selimhanay@bilkent.edu.tr; 3 Department of Mechanical Engineering, Division of Materials Science and Engineering, and the Photonics Center, Boston University, Boston, MA 02215, USA; ekinci@bu.edu

**Keywords:** microelectromechanical systems (MEMS), nanoelectromechanical systems (NEMS), nanomechanics, transducer, actuator, sensor, optomechanics, electromechanics, oscillator, resonator

## Abstract

Reliable operation of a miniaturized mechanical system requires that nanomechanical motion be transduced into electrical signals (and vice versa) with high fidelity and in a robust manner. Progress in transducer technologies is expected to impact numerous emerging and future applications of micro- and, especially, nanoelectromechanical systems (MEMS and NEMS); furthermore, high-precision measurements of nanomechanical motion are broadly used to study fundamental phenomena in physics and biology. Therefore, development of nanomechanical motion transducers with high sensitivity and bandwidth has been a central research thrust in the fields of MEMS and NEMS. Here, we will review recent progress in this rapidly-advancing area.

## 1. Introduction

A miniaturized mechanical system or a mechanical resonator [1,2] is based on the motion of a tiny solid element, typically in the form of a cantilever, a doubly-clamped beam or a torsion pad. The mechanical device responds to external perturbations and input signals by stretching, bending or twisting. The overarching characteristic of this nanomechanical motion is that it usually takes place at a high frequency with an extremely small amplitude. Depending on the relevant linear dimension, the generic names for these classes of miniaturized devices are micro- and nanoelectromechanical systems (MEMS and NEMS), with the understanding that nanomechanical motion is typically actuated and sensed in the electrical domain. Most initial MEMS were fabricated out of semiconductors and metals [3]. As linear dimensions are being pushed deep into the nanometer scale, the NEMS community is exploring other materials [4,5], including molecular materials [6,7] such as graphene, in addition to silicon [8] and Gallium Arsenide [9].

Our aim in this article is to provide a review of nanomechanical motion transducers for miniaturized mechanical systems, i.e., MEMS and NEMS. Some previous reviews *do* exist both for MEMS [10,11] and NEMS [2], but this is a rapidly advancing sub-field with contributions from different disciplines. We will not limit this review to either MEMS or NEMS. The common theme in all the reviewed work will be the transduction of nanoscale motion (or nanomechanical motion), regardless of the size of the mechanical system. We will attempt to focus on the developments in the past few years in an effort to remedy the outdated aspects of the previous reviews. We will highlight scalable techniques that may offer promise for future NEMS. The emphasis of the review, therefore, will tend to be on NEMS.

### 1.1. Basic Parameters

A miniaturized resonator oscillating in one of its modes can be modeled as a one-dimensional damped harmonic oscillator under a time-dependent driving force F(t):

(1) $$\ddot{x}+\gamma \dot{x}+{\left(2\pi f_{r}\right)}^{2}x=F\left(t\right).$$

Here, x(t) is the modal coordinate, $f_{r}$ is the resonance frequency of the mode, and γ is the dissipation — typically, described in terms of the quality factor, $Q=2\pi f_{r}/\gamma$. The dissipation experienced by a vibrating nanostructure can come from a variety of sources. Some well-studied examples include clamping losses, thermoelastic damping, and damping from surrounding fluids [12,13,14,15,16,17,18,19,20]. Since dissipation (*Q*-factor) is an important parameter in both fundamental and engineering aspects of miniaturized resonators [21], there have been attempts to control and enhance *Q*-factor, for example, via chemical surface treatment [22], external circuits [23] and parametric amplification [24]. The resonance frequency $f_{r}$ is determined by the geometry—e.g., cantilevered or torsional structures—and the linear dimensions of the device as well as the device material. The stress field within the structure, either intrinsic or induced externally, also affects $f_{r}$ and γ, and can thus be used for tuning [25,26,27].

### 1.2. Operation and Transducers

The operation and applications of miniaturized mechanical resonators are essentially governed by Equation (1) above, requiring in general three transduction elements or transducers [1]. A transducer converts energy from one physical domain to another. The input transducer converts an electrical or optical signal into a force F(t) that causes the motion; this is the *motion actuator*. Conversely, the output transducer converts the mechanical motion into an electrical or optical modulation that can eventually be detected in the time or the frequency domain; this is the *motion (displacement) sensor or detector*. The third transducer, the *control transducer*, typically relates to the interaction of the mechanical structure with its environment. In applications and experiments so far, the control transducer has been thought to be the external perturbation that changes the mechanical parameters of the device and leads to detectable output changes. In nanomechanical sensing, the performance of the control transducer is often quantified by a responsivity, which relates some observed change in the resonator’s response to the magnitude of the perturbation. Examples of perturbations (in the form of signals) come from physical, biological, or biochemical domains: the extra charge from a single electron [28], the mass of a single adsorbed molecule [29], the random motion of adsorbed bacteria [30], the added dissipation from a fluid [31], turbulent pressure fluctuations [32], and so on.

In order to explore these external perturbations ingrained in nanomechanical motion, advances are needed in techniques for actuation and detection of nanomechanical motion. Two of the most crucial attributes of a good transducer are its sensitivity and bandwidth. In other words, the sensor must provide enough response so that nanomechanical displacement can be distinguished from noise; similarly, the actuator must generate a sufficiently large force in order to excite detectable motion in the presence of other forces; and both must come with high temporal resolution for detecting or generating rapid oscillations. Some order-of-magnitude estimates may help the reader to comprehend the challenges in nanomechanical transducer development. Let us consider a silicon nanocantilever with linear dimensions of *l* × *w* × *t* = 2.5 μm × 250 nm × 250 nm. Based on the Young’s modulus *E* and density ρ of silicon, this nanocantilever will have an effective mass $m^{*}\approx 0.24\rho lwt\approx 90$ ag ($1\;\mathrm{ag}=10^{-18}\;g$) and an effective spring constant $\kappa^{*}=4\pi^{2}{f_{r}}^{2}m^{*}=Et^{3}w/4l^{3}\approx 10$ N/m [33]. The corresponding resonance frequency is $f_{r}=\left(1/2\pi \right)\sqrt{\kappa^{*}/m^{*}}\approx 60$ MHz. The thermomechanical displacement noise spectral density $S_{th}\left(f\right)$ at $f=f_{r}$ provides a good measure of the required displacement sensitivity and can be estimated as $S_{th}\left(f_{r}\right)=4k_{B}T/\kappa^{*}\gamma$, where $k_{B}T$ is the thermal energy [34]. The thermomechanical displacement fluctuations are generated by a fluctuating thermal force with frequency-independent (white) spectral density $S_{F}\approx 4\gamma k_{B}T$; $S_{F}$ provides a good scale which the actuator could be compared to. For this nanocantilever, $\sqrt{S_{th}\left(f_{r}\right)}∼0.2$ pm/Hz${}^{1/2}$ and $\sqrt{S_{F}}∼0.2$ fN/Hz${}^{1/2}$ at room temperature assuming γ ∼ $4\times 10^{4}$ s${}^{-1}$, which is equivalent to a quality-factor $Q∼10^{4}$ and is reasonable for high vacuum operation [35]. In atmospheric pressure [18,19], $Q∼10^{2}$, and $\sqrt{S_{th}\left(f_{r}\right)}∼0.02$ pm/Hz${}^{1/2}$ and $\sqrt{S_{F}}∼2$ fN/Hz${}^{1/2}$. In a liquid buffer [30,31], *Q* further goes down to Q∼ 1–2, resulting in $\sqrt{S_{th}\left(f_{r}\right)}∼3$ fm/Hz${}^{1/2}$ and $\sqrt{S_{F}}∼16$ fN/Hz${}^{1/2}$. It can be seen from these estimates that attaining extremely low noise is a must for any nanomechanical displacement sensor; motion actuators, on the other hand, have less stringent requirements. Other attributes, such as arrayed operation, robustness, ease of fabrication, and integration with current electrical/optical measurement methodologies, are also desirable. In our discussion below, we will attempt to evaluate some of the emerging techniques using these basic criteria.

## 2. Actuation of Nanomechanical Motion

In this section, we describe some recently-developed nanomechanical motion actuators in the MEMS and NEMS domains. For convenience, we arbitrarily divide the discussion into two subsections. We first focus on actuation techniques based solely on light. We then touch upon techniques based on electronic coupling.

### 2.1. Optical Techniques

**Figure 1:**
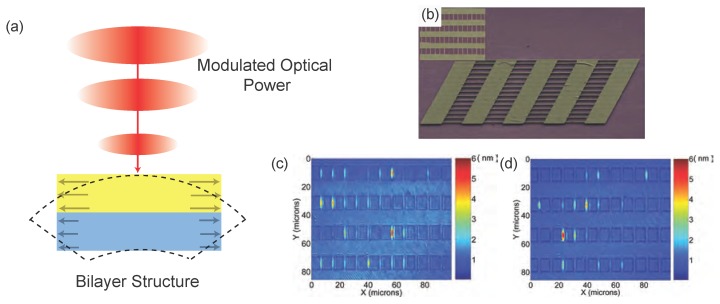
Photothermal actuation of nanoelectromechanical systems (NEMS). (**a**) The optical power incident on the NEMS is intensity modulated at the frequency of actuation. The bilayer structure heats up and cools down around a mean temperature at the same frequency. Thermal stresses are developed, e.g., due to the different thermal expansion coefficients of the layers. As a result, the structure moves; (**b**) scanning electron microscope (SEM) image of an array of nanomechanical beam resonators with linear dimensions l×w×t= 10 μm ×1μm ×200 nm from Sampathkumar et al. [37]; (**c**,**d**) colormaps corresponding to the out-of-plane displacements of a 48-element NEMS array measured by a full-field optical interferometer. The array was excited sinusoidally at 21.5 MHz (**c**) and 22.0 MHz (**d**). The bright spots correspond to elements which move appreciably due to mechanical resonances at these frequencies. The fundamental resonance frequencies are not identical, even though all the linear dimensions (l×w×t) are, probably because of the tolerances of the various fabrication steps. Such dispersions are not uncommon in the NEMS domain. Reprinted with permission from ref. [37]. Copyright 2011 American Chemical Society.

The simplest and perhaps most explored physical mechanism by which light can exert forces on a mechanical resonator is the photothermal effect [36]. Here, one relies on the periodic heating of a mechanical element by a modulated optical field, as shown in Figure 1a. Assuming a sinusoidal optical modulation, the optical power absorbed by the mechanical element results in a temperature increase during the first half of the cycle; the element subsequently cools in the second half of the cycle.

Thermal stresses generated in the device then excite nanomechanical motion (Figure 1a). This actuation method works most efficiently in micro- and nanostructures made of layers of different materials with different thermal expansion coefficients. One undesirable aspect here is that the mechanical structure remains at a mean temperature higher than the surrounding bath. The thermal relaxation time of the mechanical resonator determines the bandwidth of the actuator, and the technique tends to become less efficient at high frequencies. For instance, the thermal relaxation time for the silicon nanocantilever example of Section 1.2 is estimated to be ∼100 ns, suggesting that photothermal actuation would not generate sufficient force above 10 MHz for a structure like this. It is straightforward to actuate NEMS photothermally using free-space optics, but the technique could also be implemented within an integrated optical chip. Using a tightly focused light beam results in high spatial resolution for the photothermal actuator, allowing for the actuation of higher modes of NEMS. Conversely, arrays of NEMS have been actuated by expanding the optical spot [37], as shown in Figure 1b–d. We note that actuation based on periodic heating can also be accomplished electronically as described below in Section 2.2.

**Figure 2:**
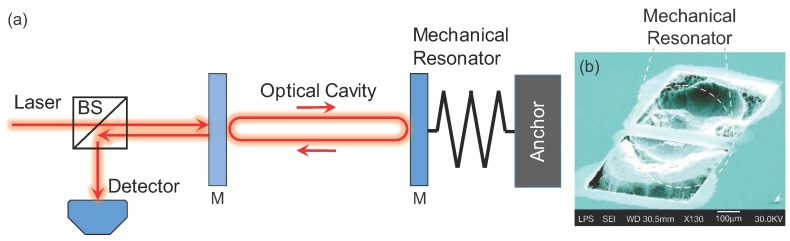
An optomechanical system and actuation of nanomechanical motion by radiation pressure. (**a**) The optomechanical system is comprised of two mirrors; one of these (right) is attached to a spring and is the mechanical resonator. The two mirrors together form an optical cavity in which an optical field circulates. In this configuration, light exerts radiation pressure on the movable mirror. The optical and the mechanical degrees of freedom are coupled due to the motion of the mechanical resonator; (**b**) a highly reflective micromechanical resonator fabricated by Gigan et al. [38]. This mechanical resonator formed the movable mirror in an optical cavity such as that shown in (**a**), and the radiation pressure in the cavity efficiently actuated the motion of this mechanical resonator. Reprinted by permission from Macmillan Publishers Ltd.: Nature [38], copyright 2006.

Radiation pressure has been used for exerting controllable forces on miniaturized mechanical resonators. Here, photons incident on a reflective surface bounce back but transfer momentum to the surface, thus creating a push force called radiation pressure [38,39,40,41,42,43,44]. Radiation pressure has previously been exploited for manipulating and trapping micro- and nanoparticles in optical traps. One of the challenges in using radiation pressure for actuating miniaturized mechanical systems is the typically low reflectivity of the device surface due to diffraction, optical mode mismatches, and other factors. In order to increase the efficiency of radiation pressure actuation, researchers have explored highly reflective mechanical structures, such as miniaturized mirrors, Bragg reflectors (Figure 2b) and diffraction gratings [45]. Because making highly-reflective miniaturized structures remains a challenge, most actuators based on radiation pressure are in the MEMS domain. Nevertheless, some of the recent work aiming to attain the ground state of a mechanical resonator has benefited from radiation pressure since optical absorption can be reduced here effectively—as opposed to photothermal actuation.

**Figure 3:**
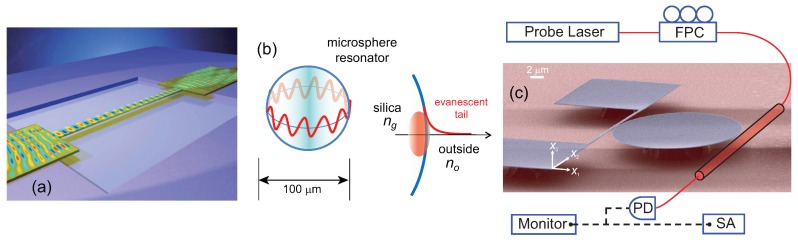
Optical gradient forces for actuating nanomechanical motion. (**a**) Suspended waveguide nanobeam showing the optical mode plot within the waveguide [60]. By modulating the optical intensity, optical gradient forces are generated on the suspended nanobeam and pull the beam toward the substrate. Reprinted by permission from Macmillan Publishers Ltd.: Nature [60], copyright 2008; (**b**) illustration of the whispering gallery mode (WGM) optical resonances of a silica microsphere and the field amplitude around the surface (right). The refractive indices of the microsphere and the surrounding (e.g., air or water) are $n_{g}$ and $n_{o}$, respectively. The microsphere is similar to the mirror arrangement in Figure 2a in that the optical field circulates around the microsphere and is enhanced by resonances. The field is localized on the surface of the microsphere, but an evanescent tail extends into the surrounding environment. The optical resonances of the microsphere can be coupled to mechanical structures and modes by virtue of these evanescent tails. Alternatively, the mechanical modes of the sphere itself can serve as the mechanical resonator coupled to the optical field; (**c**) a nano-opto-mechanical device by Basarir et al. [65] comprised of a nanomechanical beam resonator and a microdisk optical resonator. The linear dimensions of the beam are l×t×w=15μm×230nm×250nm and the disk diameter is 20 μm. An external tapered fiber is used for optical actuation and detection. Photodetector: PD; fiber polarization controller: FPC; a spectrum analyzer (SA) is used for the measurements. Here, the gradient force pulls the nanobeam toward the disk (in the $x_{1}$ direction). If the optical intensity is modulated, mechanical motion is actuated at the modulation frequency. The optical miscrodisk resonator stores optical power and increases the efficiency of the actuator significantly. Reprinted with permission from ref. [65]. Copyright 2011 The Optical Society (OSA).

Both radiation pressure based actuators and other optical actuators have been implemented efficiently within optical cavities. There are authoritative reviews on optical cavities and the interested reader can consult these [46]; we provide here a very brief introduction to optical cavities in the context of micro- and nano-optomechanics. An optical cavity enhances the optical field by means of constructive interference, as shown in Figure 2a. Ignoring for the moment the mechanical degree of freedom in the mirror arrangement in Figure 2a, one notices that light waves that enter the cavity undergo multiple reflections at the mirrors, thus creating a “circulating optical field” inside the cavity. This circulating field (and the energy stored in the cavity) can be many orders of magnitude larger than the steady-state energy input (per cycle) into the cavity, if the cavity length is such that constructive interference is facilitated. The quality of the cavity, e.g., the quality of the mirrors in Figure 2a, is also of importance for attaining large optical fields inside the cavity. The mirror arrangement shown in Figure 2a is the well-known Fabry–Perot cavity (Fabry–Perot etalon) of macroscopic optics; researchers have exploited whispering gallery mode (WGM) optical resonances in microspheres [47,48], microdisks [49,50], microtoroids [51] and photonic crystal microstructures [52,53,54] to create optical cavities in the microscopic domain. (A microsphere is shown in Figure 3b.) These devices are referred to as optical cavities, optical resonators, microcavitites or WGM resonators.

Returning to Figure 2a, we notice that one of the mirrors is attached to a spring, coupling the optical field to the mechanical degree of freedom. As the movable mirror oscillates back and forth, the optical path length in the cavity changes, giving rise to a change in the optical resonance condition. The end result is a change in the circulating field, which, by virtue of radiation pressure, exerts a force on the movable mirror [38,39,40,41]. The complex mutual coupling between the mechanical and optical degrees of freedom is referred to as optomechanics in general and micro- or nano-optomechanics [55,56] in the case of miniaturized mechanical devices. Naïvely, by virtue of the stored energy, an optical cavity can be thought to enhance the interaction between the mechanical resonator and the optical field—provided that the two are coupled efficiently. This increased interaction then results in stronger actuation forces or detection signals (see Section 3.2 below). Furthermore, one can exploit optical forces in an optical cavity (i.e., the optomechanical coupling) in order to obtain interesting dynamical phenomena, including feedback to “cool” (attenuate) or amplify the nanomechanical oscillations.

Another commonly-used optical actuator is based on optical dipole forces or optical gradient forces [57], which originate from field gradients or field inhomogeneities. This type of force has previously been used for the manipulation of microparticles in optical traps, where field gradients are formed within a tightly-focused laser spot [58]. The gradient force actuator is readily scalable into the NEMS domain, where researchers have implemented gradient force actuators by exploiting the field gradients around optical waveguides. As such, the approach appears suitable for optically integrated transducers and has allowed for progress toward integrated nanooptoelectromechanical systems (NOEMS) [59,60,61]. Briefly, optical or photonic integration refers to the concept of confining light on a planar semiconductor chip. Light waves travel inside “circuits” formed from waveguides and other components fabricated on a chip—as opposed to traveling in free space. Materials, such as silicon, which have high refractive indices, are suitable for optically-integrated MEMS and NEMS (and other devices), because they allow for the light to be confined in small structures. Figure 3a shows a waveguide-based nanomechanical structure [60]. Here, optical waves travel mostly inside the suspended waveguide, which can be “pulled” toward the substrate using gradient forces if the optical intensity is modulated properly. Optical gradient forces can be used to actuate miniaturized structures in other geometries, for instance, by using a secondary waveguide brought in the vicinity of the structure [62,63,64]. As in radiation pressure, the use of optical cavities or resonators can increase the magnitude of the applied forces significantly [65,66]. As shown in Figure 3c [65], an on-chip optical resonator can be fabricated (or positioned) in the vicinity of a nanomechanical resonator; if the optical field is modulated properly, mechanical motion can be actuated at the modulation frequency.

Radiation pressure and gradient forces can in principle be used to actuate nanostructures at very high frequencies, provided that the optical intensity can be modulated efficiently. Optically integrated transducers can also ease some of the stringent alignment requirements of free-space optics. As we discuss below in Section 3.2, motion detection can also be accomplished in optically-integrated mechanical structures by exploiting a variety of optical phenomena, enabling fully integrated micro- and nanooptomechanical systems (MOMS and NOMS).

### 2.2. Electronic Coupling

Here, we touch upon some recently developed electronic actuation techniques, keeping an eye on scalability. Early experiments used magnetomotive actuation in which an alternating current (AC) passing through a nanobeam placed in a static magnetic field creates a Lorentz force [8]. Unfortunately, the magnetic fields required to obtain a sufficiently strong force is very large: it is usually necessary to use superconducting magnets cooled with liquid Helium. Moreover, the magnetomotive technique is inefficient in actuating higher modes—which are essential for sensing applications [29,67,68,69] and mechanical mode coupling experiments [70,71,72,73,74]—since the total force integrated along the higher modeshapes almost cancels out. Piezoelectric ceramic discs (shakers) have also been used extensively to actuate NEMS and MEMS (e.g., microcantilevers) chips placed on top; however, this is not an integrated and efficient scheme, and the actuation linearity becomes severely degraded at high frequencies. Therefore, exciting higher order modes of a miniaturized mechanical structure with integrated electrodes have been one of the major research goals in the pursuit of electronic actuators.

**Figure 4:**
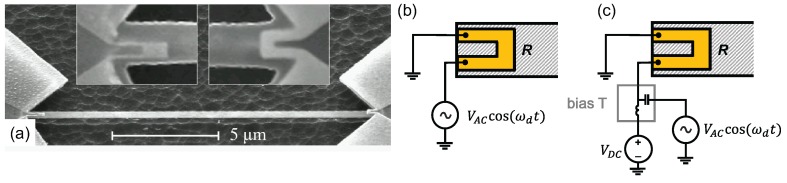
(**a**) SEM image of a device used for thermoelastic actuation and piezoresistive detection. The nanomechanical doubly-clamped beam is made out of SiC, and the metallic electrodes are fabricated on both sides. Voltage applied to one side generates stress. In this device, the right electrode was optimized for actuation. The readout signal is obtained from the other electrode which exhibits geometric piezoresistivity, as discussed below in Section 3.3. Reprinted from [75] with permission of AIP Publishing; (**b**) circuit diagram for thermoelastic actuation in alternating current (AC)-only mode; resonance condition is reached when $\omega_{d}=\omega_{m}/2$, where $\omega_{d}$ is the drive frequency and $\omega_{m}$ is the mechanical resonance frequency; (**c**) circuit diagram for thermoelastic actuation in AC + direct current (DC) mode; resonance condition is reached when $\omega_{d}=\omega_{m}$.

One of the first demonstrations of an integrated actuation scheme, which can access higher order modes, is thermoelastic actuation [75], shown in Figure 4. In this technique, metallic electrodes are fabricated on one end of a flexural resonator. The electrodes are designed to have a relatively large resistance (compared to the rest of the metalic path) so that any applied voltage creates a localized Joule heating on the resonator. Local heating of the metallic electrode and the underlying mechanical structure causes both materials to expand. However, the expansion coefficients of these materials are different: as a result a stress that is modulated by the applied voltage develops. Since heating is proportional to the square of the voltage, mechanical actuation takes place at twice the electrical input frequency. In this way, the technique resembles photothermal actuation discussed in Section 2.1. Thermoelastic actuation requires two different materials with different thermal expansion coefficients. There are two different modes of operation: in AC-only operation, a single AC signal at half the mechanical resonance frequency is applied; in AC + direct current (DC) operation, a combination of a DC biasing voltage and an AC voltage at the resonance frequency drives the structure. The second technique is especially useful if the measurement needs to be done with a network analyzer, since AC actuation and mechanical detection frequencies become identical. Moreover, since electrodes are made of metal, it is possible to fabricate electrodes with resistances near 50Ω, enabling an efficient coupling to radiofrequency (RF) electronics.

**Figure 5:**
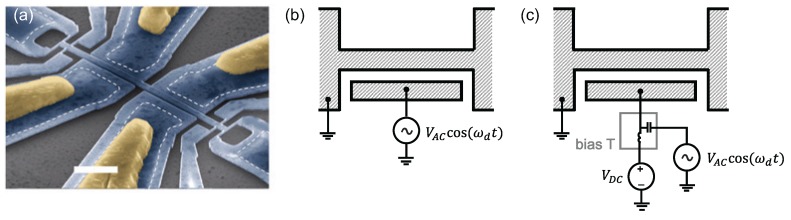
(**a**) An example of a silicon NEMS device actuated electrostatically and detected using piezoresistive gauges. Silicon is shown in blue and the metallic contacts in yellow. White dotted lines indicate the boundary between suspended and unsuspended regions. Four gates that can be used for in-plane actuation are fabricated near the central resonator structure. The suspended small bridges on each end of the structure work as piezoresistive gauges. Reprinted by permission from Macmillan Publishers Ltd.: Nature Nanotechnology [29], copyright 2012; (**b**) circuit diagram for capacitive actuation with AC-only mode. Resonant motion is driven when $\omega_{d}=\omega_{m}/2$; (**c**) circuit diagram for capacitive actuation with AC + DC mode. Resonant motion is driven when $\omega_{d}=\omega_{m}$. Both modes produce a static deflection of the beam due to a DC-term in the force expression.

Capacitive or electrostatic actuation is a commonly used technique, especially for MEMS devices. In the MEMS domain, comb drive designs with multiple interdigitated electrodes have been used for linear motion actuation based on electrostatic forces. Although several fundamental studies have been performed in such larger MEMS devices actuated by comb drives, such as frequency stabilization through Internal Resonances [76], comb drives are hard to fabricate and increase the effective mass of a resonator—a disadvantage for typical sensor applications. In the NEMS domain, electrostatic actuation is usually achieved with a simple side gate [77]. The gate electrode fabricated near the resonator generates a driving force as shown in Figure 5. The force can be calculated by taking the derivative of the electric potential energy *U* with respect to the motion coordinate *x*: $F_{x}=-\partial U/\partial x=-C^{\prime }\left(x\right)V^{2}$, where C(x) is the position dependent capacitance and *V* is the voltage across this capacitance. As with thermoelastic actuation, the square of the applied voltage determines the mechanical actuation force. When the resonator is driven at large amplitudes using a simple gate geometry, the actuation force exhibits softening nonlinearity which is a hallmark of capacitive actuation. Fundamental physics experiments at low temperatures probing the ultimate limits of position detection [78] and cooling nanoscale resonators through back-action [79] used such capacitive gates to drive the resonator. Capacitive drive at room-temperature has also been used to actuate in-plane flexural motion of clamped-clamped beams [80] and pinned-pinned beams [29,81] in practical applications. Capacitive actuation has been especially useful in carbon nanotube [82] and graphene [6] nanomechanical devices since the entire substrate, over which the structure is suspended, can be conveniently used as the gate electrode. However, this approach increases the parasitic capacitance significantly. An elaborate solution is to fabricate a local gate at an earlier lithography stage [83]. In this work [83], the mechanical resonance was measured without the down-mixing technique which retains the full bandwidth of the resonator for use in potential RF filter and oscillator applications.

A related technique is reported in [84], where fringing electric fields induce parametric excitation on a silicon-on-insulator (SOI) cantilever. In this device, the electrode and the cantilever are located on the same plane: by modulating the effective stiffness of the cantilever, out-of-plane oscillations are generated. Since the oscillation direction is perpendicular to the gap between the cantilever and the electrode, some of the problems seen in direct electrostatic actuation, such as the pull-in instability and stiction, are avoided in this technique.

**Figure 6:**
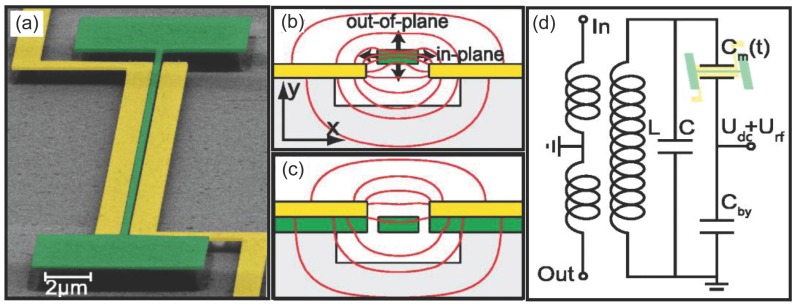
(**a**) Micrograph of a device used in dielectric gradient actuation. A doubly-clamped beam made of silicon nitride (green) is placed between two yellow electrodes made of gold. No metallization layer is needed on the resonator. The dielectric structure moves toward the region of larger electric field. If asymmetric voltages are applied on the two electrodes, in-plane motion can be induced. If symmetric voltages are applied, out-of-plane motion can be induced since electric field distribution has higher density on a plane above the resonator, as shown for the case where (**b**) metal layers are below the resonator and (**c**) metal layers are placed above the resonator; (**d**) circuit diagram showing how to combine dielectric gradient force actuation with microwave optomechanical readout on the same device. An LC tank formed by a microstripline is connected to the nanoresonator via wirebonds. By using a bypass capacitor to form a high-frequency ground for the GHz range microwave tank but an open circuit for the MHz frequency microwave driving signal ($U_{dc}+U_{rf}$), it is possible to combine both detection and actuation functions on one of the electrodes. Reprinted from [87], with the permission of AIP Publishing.

In electrostatic actuation discussed above, both the gate electrode and the resonator structure need to be conductive or have an additional metallic layer so that the desired voltage difference can be applied. A different technique based on the inhomogeneities of the electric field obviates the need for a metallic layer on the structure (Figure 6). The technique utilizes electric dipole forces generated when a dielectric material is placed in an inhomogeneous electric field [85,86]. The dielectric beam is attracted to the region with the larger electric field. In this way, the technique uses a principle similar to the optical gradient forces mentioned in Section 2.1. In the first demonstration [85], a silicon nitride beam was placed near gold electrodes that were biased in the AC + DC manner to generate the inhomogeneous AC electric field. Since there is no need to deposit additional material on the resonator (unlike in the electrostatic or thermoelastic techniques), structural damping due to the elastic mismatch between layers vanishes, enabling very high *Q* factors: $Q∼10^{5}$ in [85] and $Q∼3.4\times 10^{5}$ in [87]. This dielectric gradient force technique has been used to actuate single-crystal diamond nanocantilevers and doubly-clamped nanobeams [86] as well. In both examples, the electrodes were placed on the substrate after the suspended nanostructures were fabricated. With this technique, the frequency of the resonator can be tuned by the applied voltage, enabling parametric amplification when an AC signal at twice the mechanical resonance is applied on the electrodes.

The actuation principle based on electric field inhomogeneities can be reversed to detect mechanical motion. In this case, a different set of electrodes is used to measure the electrical modulation due to the resonator motion. In order to avoid cross-talk from the input electrical signal, parametric excitation is used so that the actuation takes place at twice the mechanical resonance frequency and the detection at the resonance frequency. Moreover, by applying voltages asymmetrically to two adjacent electrodes, it is possible to actuate the in-plane motion in this technique [87]. In this study, the DC voltage was used not only to tune the resonance frequency but also to decrease the *Q* by a factor of 6—a feature that could be useful in high-speed atomic force microscopy (AFM) [88].

Piezoelectric actuation has been commonly used in MEMS devices with micron-scale piezoelectric layers. Piezoelectric effect can be described by the piezoelectric constant $d_{ij}$ which is the ratio of the strain in the *j*-direction to the electric field in the *i*-direction (hence, its unit is m/V). Higher values of $d_{ij}$ correspond to more efficient actuation in the sense that a given voltage produces a larger deformation. Since piezoelectricity enables a direct conversion between electrical signals and mechanical deformation, it is an appealing technique for transduction. However, it was unclear whether the technique could be scaled down to submicron thicknesses as the crystalline orientation had to be preserved during the deposition process. Initial experiments that accomplished piezoelectricity at nanoscale thicknesses used gallium arsenide (GaAs) grown by molecular beam epitaxy (MBE) [89]. In this case, a three-layer stack with a total thickness of 200 nm was fabricated. The resonator structure had *n*-doped, intrinsic and *p*-doped GaAs layers. The electric field across the intrinsic region, imposed by the charge on the depletion layers, generates a stress through the $d_{31}$ coefficient of GaAs. The actuation voltage was applied between a gold electrode fabricated at the top of the stack and the degenerately doped substrate through a metallic contact at the bottom of the chip. With an electric field perpendicular to the structure, piezoelectric $d_{31}$ coupling generates an axial stress on the stack. To convert the axial stress into a bending moment, the piezoelectrically active intrinsic GaAs layer is placed asymmetrically in the stack. This design rule, i.e., the placement of the piezo-active stack away from the neutral axis of the beam, has been adapted to other piezoelectric resonators. In this work [89], the piezoelectric constant was measured to be $d_{31}=-1.33\;\mathrm{pm}/V$.

**Figure 7:**
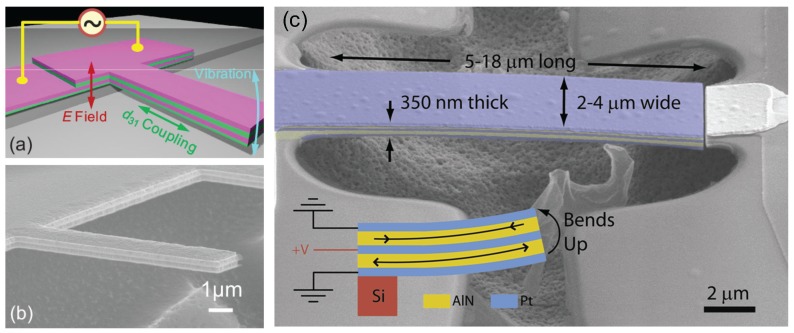
Piezoelectric actuation. (**a**) In most experiments using piezoelectric actuation at the nanoscale, the $d_{31}$ coupling is used. Here, a voltage difference is applied between the electrode layers sandwiching the piezoelectric material. Resulting electric field in the *z* direction creates an axial strain along the *x* direction. This axial strain causes a bending moment which excites the flexural motion, if the piezoelectric layer is asymmetric with respect to the neutral axis [5]; (**b**) SEM micrograph of the four layer device (AlN/Mo/AlN/Mo) fabricated in [5]. Reprinted from [5], with the permission of AIP Publishing; (**c**) five layer (Pt/AlN/Pt/AlN/Pt) device fabricated in [90]. By keeping the top and bottom Pt electrodes at ground and applying a voltage to the center Pt electrode, bimorph actuation is possible. Reprinted from [90], with the permission of AIP Publishing.

After the work of Masmanidis et al. [89], several studies [5,90] focused on using aluminum nitride (AlN) as the piezoelectric material, since thin piezoelectric AlN films can be readily sputtered. In one process [91], sputtering of Molybdenum films was optimized so that additional AlN deposited on top would possess a definite *c*-axis and exhibit piezoelectricity. In this case, the Molybdenum layer also provided a metallic contact for applying the voltage. Thus, a piezoelectric device with a 100-nm AlN layer was fabricated, as shown in Figure 7a,b [5]. The AlN layer here is placed between two 100-nm Molybdenum layers for electrical contact; an additional 20-nm AlN layer was placed at the bottom to displace the middle AlN layer away from the neutral axis. This device was actuated piezoelectrically, and its motion was detected by optical interferometry. The $d_{31}$ constant was measured to be 2.4pm/V. Another work around the same time accomplished the sputtering of a 100-nm piezoelectric AlN film [90]. Devices with five layers of Platinum and AlN (Pt/AlN/Pt/AlN/Pt) that enabled bimorph actuation as shown Figure 7c were fabricated. In this work, only the static deflection of the mechanical structure was measured using optical interferometry. The reported piezoelectric coefficient, $d_{31}=1.9\;\mathrm{pm}/V$, is slightly smaller than the aforementioned AlN work.

Piezoelectric coupling also offers a possibility for parametric actuation through the modulation of the effective stiffness of the structure. Parametric amplification has been demonstrated with the systems mentioned above, e.g., the GaAs system [92] and the AlN system [93]. Another line of work used GaAs/AlGaAs system to excite and detect motion using piezoelectric electrodes on both sides of a doubly-clamped beam [94]. In this case, a small harmonic actuation signal (at ω) is applied to one electrode loop and a parametric pump is applied (at 2ω) to another one: the resonance is detected through the inverse effect on the third electrode. A parametric oscillator (without harmonic actuation) was accomplished in a follow up work [95] where the effective linewidth of resonance was reduced more than 1000-fold due to the parametric drive.

Finally, coupling a nanomechanical structure to a microwave resonator can also be used to actuate mechanical motion by ”blue-detuning” the microwave resonator by an amount equal to the mechanical resonance frequency [87,96]. Since this technique is inherently connected to detection, a detailed discussion is deferred to Section 3.3 on electrical detection.

## 3. Detection of Nanomechanical Motion

We now turn to motion detection techniques or displacement sensors. Again, we have arbitrarily divided our review into optical techniques and techniques not involving light. Furthermore, we try to differentiate between integrated and free-space optical techniques—even though some transducers combine the two and are thus difficult to categorize as either. As before, the emphasis will be on the scalability of the techniques discussed.

### 3.1. Techniques Based on Free-Space Optics

**Figure 8:**
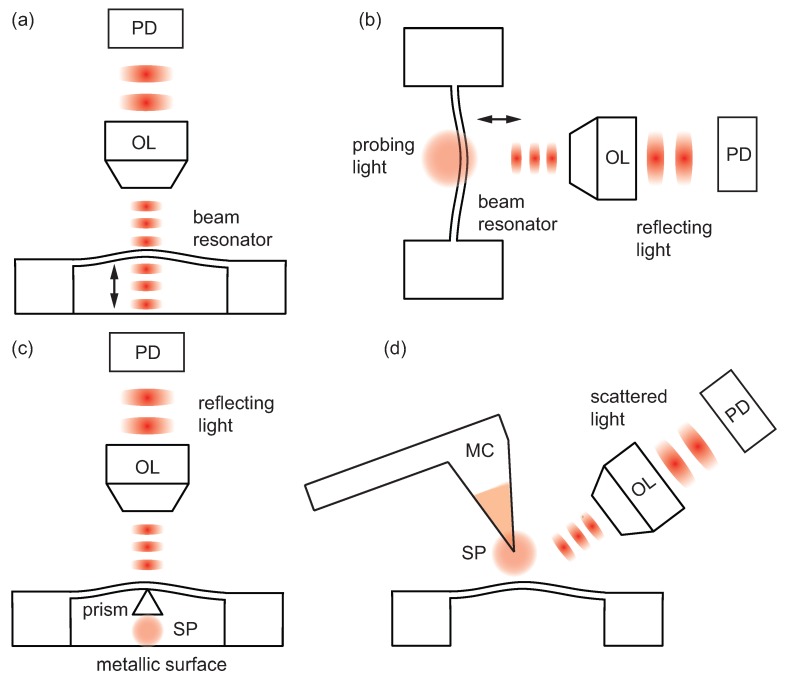
Nanomechanical motion detection techniques based on free-space optics. (**a**) Fabry–Perot interferometer with multiple reflections of light between a doubly-clamped nanoscale beam resonator and the underlying substrate, focused with an objective lens (OL) and measured by a photodetector (PD); (**b**) optical knife-edge technique. The probing light is focused normally onto a beam resonator moving in-plane, and the light reflecting from the surface to PD is monitored; (**c**) near-field optical motion detection using a plasmonic structure as reported in [112]. Surface plasmons (SP) are excited within the dielectric gap between the NEMS resonator and the metallic surface underneath via a prism embedded in the NEMS resonator. The reflected optical field modulated by the motion of the prism along the beam is measured by a PD at the far field; (**d**) near-field optical motion detection using a metallic tip on a microcantilever (MC) as in [113]. The external light excites surface plasmons confined at the sharp tip, and the change in the scattered light intensity from the tip due to the vibration of the resonator is monitored at the far field.

The mainstay free-space optical approach for transducing the mechanical motion of miniaturized mechanical systems has been optical interferometry. With stable laser sources, and fast and sensitive photodetectors, interferometry provides a very high displacement sensitivity and a large measurement bandwidth, and is suitable for room-temperature applications. In a simple Michelson type interferometer, one interferes the optical beam reflecting from the surface of the miniaturized resonator with a reference beam. A Fabry–Perot type interferometer, shown in Figure 8a, uses multiple reflections of the same optical beam and tends to increase the sensitivity. Optical interferometry has served the MEMS and NEMS communities well in early and ongoing works [97,98,99,100,101,102,103]. Recently, interferometry has been further exploited to read out the motion of an array of NEMS resonators [37]. This adaptive full-field interferometer successfully mapped out the photothermally-induced individual mechanical resonances of multiple doubly-clamped beam resonators—about 40 beam resonators—within a ∼ 100 μm × 100 μm area, as shown in Figure 1. In another recent work, the oscillations of Si nanowire resonators with widths between 100–200 nm were detected by monitoring the interference between the leaky optical resonance modes around the wires and the surrounding electromagnetic field from the substrate within a Fabry–Perot-like cavity formed by the nanowires and the substrate underneath [104].

In interferometry, light is typically tightly focused on the device using an objective lens, and one cannot achieve an optical spot size below the diffraction limit. This significantly degrades the displacement sensitivity as the linear dimensions of a NEMS device becomes smaller than the diameter of the optical spot, which is typically around a few micrometers. Second, the motion of the device must be along the optical path (in the out-of-plane direction) since this motion generates a change in the optical path length required for interferometry. These factors complicate optical interferometry (as well as other optical techniques) that are based on free-space optics.

Under certain circumstances, one might need to detect degrees of freedom of mechanical motion other than those moving along the optical path. For instance, if one actuates a mechanical structure in the in-plane direction, interferometric techniques tend to become insensitive to the mechanical motion. Recently demonstrated transduction techniques suitable for such motions are primarily based on optical scattering: optical knife-edge technique [105,106,107], and scattering enhanced with near-field optical probes and/or approaches [59,63,108,109,110,111,112,113,114].

The optical knife-edge technique for in-plane motion of NEMS [105] is in principle similar to the optical deflection detection method for a microcantilever using a sectioned photodiode or a knife-edge placed in front of a regular photodiode [34,115]. In the optical knife-edge technique, the device to be probed works as a knife-edge, and its motion modulates the optical signal reflecting toward the photodetector, as shown in Figure 8b. The displacement sensitivity of this approach for a doubly-clamped beam resonator can be estimated by carefully scanning the optical spot along the width of the beam and monitoring the resulting reflected optical signal at each step, as long as the beam length is sufficiently long so that the bending of the beam within the optical spot is negligible. The demonstrated displacement sensitivity at sub-mW power levels is around 1 pm/Hz${}^{1/2}$ for a subwavelength doubly-clamped beam resonator. In addition to the optical power, the detection sensitivity depends on the relative sizes of the optical spot (focused by an objective lens) and the nanobeam, and is limited by diffraction—as in free-space optical interferometric approaches.

In the near-field adaptation of this technique, one exploits the interaction between the NEMS to be probed and an evanescent optical wave localized in the vicinity of the NEMS; the scattered optical power is detected by a photodetector in the far field [114]. The coupling of the evanescent wave to the NEMS resonator may be accomplished by using a variety of structures, such as a waveguide, an optical cavity, a fiber taper, a sharp metallic tip or a similar plasmonic structure. While integrated optical detection exploits on-chip components, such as a waveguide, an optical cavity or a fiber taper (see Section 2.1 and Section 3.2), free-space near-field optical motion detection employs a metallic tip or a plasmonic structure—which is the subject of this section.

The coupling of surface plasmons to suspended NEMS resonators has been achieved by plasmonic structures, such as surface plasmon-supporting metal films [110], nanoantennas [111], and prisms [112]. These plasmonic elements are embedded into the NEMS resonator or are fabricated in the vicinity of it, and form a dielectric gap with a metallic surface. The metallic surface can be in the form of a metal-coated substrate or another freestanding metallic resonator. In this way, the motion of the resonator can be directly coupled to optical modes, as shown in Figure 8c. The nanomechanical motion of the resonator changes the effective refractive index of the volume with which the optical modes interact, and the resulting optical response through the dispersive nature of the plasmonic resonance is monitored by transmission or reflection measurements at the far field. This approach has been extended to detect the motion of an array of beam resonators by using an expanded optical spot covering multiple beams separated by 20-nm-wide ion-beam-milled slits [116]. The strong spatial concentration of plasmons within the dielectric gap between the metallic surfaces, scaling with the size of the resonating device, ensures that the optomechanical coupling strength—defined as optical frequency shift per unit displacement—is above 1 THz/nm, leading to a displacement sensitivity as high as 6 fm/Hz${}^{1/2}$.

In the scanning probe microscopy (SPM)-based near-field method demonstrated by Ahn et al. [113], a sharp metallic tip on a microcantilever with a line-grating serves both as a local probe and a local source (Figure 8d). Localized surface plasmons are generated and confined at the tip, by tightly focusing the light onto the tip. The interaction between the tip and the moving device surface scatters the localized plasmons, which are then measured at the far-field with a photodetector. Because the intensity of the optical interaction varies significantly with the distance between the tip and the surface as in apertureless scattering-type near-field optical microscopy [117], the scattered light collected at the far-field carries information on the oscillations of the mechanical resonator. The reported sensitivity of this technique is about 0.45 pm/Hz${}^{1/2}$.

In these near-field approaches, the confinement of surface plasmons scales with the physical dimensions of the plasmonic structures: for the nanoantenna, the footprint is 485 nm × 50 nm; for the prism, it is 350 nm × 165 nm; and in the SPM-based approach, it is the tip radius, which is about 20 nm. This results in a detection technique which scales below the conventional diffraction limit. In some of these techniques, one can also detect the in-plane oscillations since the optical interactions only depend on the separation between the the plasmonic element on the resonator and the metallic surface or the separation between the resonator surface and the tip. For example, in the SPM-based technique, this can be done by carefully placing the tip near the side of the resonator. This capability of motion detection in both the in-plane and out-of-plane directions can provide flexibility in NEMS resonator design and fabrication. Another interesting aspect of the SPM-based method is the mechanical double-frequency demodulation. The double-frequency demodulation is performed by monitoring the optical signal at the difference frequency between the well-separated resonance frequencies of the tip-mounted microcantilever, which is typically about a few hundred kHz, and the nanoscale resonator, which is well above a few MHz. Furthermore, this can suppress the unwanted optical background noise.

### 3.2. Integrated Optical Techniques

As hinted above, near-field (evanescent) optical interactions provide an attractive avenue for nanomechanical motion transduction. Near-field optical interactions have been well explored in MEMS-scale structures [118,119]. Furthermore, they fit the length scale of NEMS well and can provide sensitivity beyond the diffraction limit. They can be used in both interferometric and non-interferometric approaches, as in free-space optical techniques. Several key elements for integrated optical motion detection, such as suspended optical waveguides and miniaturized optical cavities, have already been discussed above in optical actuation in Section 2.1; other aspects, such as use of optical scattering, are similar to far-field or free-space optical approaches that have been discussed in Section 3.1. Here, we will review some scalable approaches.

We first turn to an integrated interferometric approach [60] in which the phase of light propagating through a photonic circuit is modulated by the motion of a nanomechanical beam. Here, two on-chip waveguides are configured in a Mach–Zehnder interferometer [120]. A portion of one of the waveguides is suspended to form the nanomechanical beam resonator (Figure 3). When the waveguide (i.e., the nanomechanical beam) moves toward the substrate, the local optical index and hence the total optical path length changes. This optical path length change (phase shift) is detected in the Mach–Zender interferometer. This scheme has provided a displacement sensitivity ≤0.1 pm/${\mathrm{Hz}}^{1/2}$ at mW-level optical powers. More recently, a similar on-chip interferometric approach has been applied to detect the motion of nanocantilevers [121]. Other possibilities to generate interferometric signals, for instance, from the strain-optic (photo-elastic) effect in a flexing nanostructure, also exist but have not yet been fully explored.

Non-interferometric approaches come with less stringent coherence and stability requirements for the light. Here, one of the approaches successfully demonstrated is based on the scattering of evanescent waves in a waveguide due to nanomechanical motion [59,61,62,108,122,123,124]. In the implementation by Basarir et al. [62,125], a tapered fiber waveguide is brought in the vicinity of a NEMS resonator such that the NEMS structure interacts with the evanescent tail of the optical wave propagating through the waveguide. As the NEMS-waveguide gap is modulated due to the NEMS motion, the optical power transmitted through the waveguide is modulated due to optical scattering by the NEMS resonator. This is a simple but sensitive technique and has allowed for motion detection in arrays of NEMS resonators [63]. The demonstrated sensitivity approaches ∼0.1 pm/Hz${}^{1/2}$ at optical power levels ≤100 μW.

The detection signals in integrated optical devices described here can further be enhanced by using optical cavities or optical resonators [65,126], as in the case of optical actuation discussed in Section 2.1. By re-inspecting the device in Figure 3c, we can explain the enhancement in an intuitive manner. As the nanomechanical beam moves toward the microdisk resonator, its motion changes the local optical index of the microdisk and hence the optical path length around the microdisk, resulting in a modulation of the optical field in the microdisk. Given that the microdisk stores a large amount of optical energy at steady state, one can again naïvely assume that a small mechanical perturbation will result in a large optical response. Enhancement using a variety of cavities has commonly allowed for displacement sensitivities approaching and even below $1\;\mathrm{fm}/{\mathrm{Hz}}^{1/2}$ for different types of nanomechanical structures at mW–μW level input powers.

Finally, one of the technological challenges in developing integrated optical devices is the coupling of light into and out of the device. A commonly used technique is the direct or “butt” coupling of light [127]. In this technique, two single-mode fibers are directly aligned to the waveguides on the chip to couple the light into and out of the chip. Improvements on this straightforward technique have been achieved by using tapered couplers, lensed fibers, fiber focusers, 3D couplers, and inverse nanotapers [128]. A detailed review of some of these methods is given in [127,129,130,131]. Another approach is based on the use of grating couplers [108]; while somewhat inefficient, grating couplers are easier to implement and may be preferable in some applications.

### 3.3. Electronic and Other Approaches

The widespread electronic displacement detection techniques of the MEMS domain, e.g., capacitive detection, do not scale into the NEMS domain in a straightforward manner (see below). As with electronic actuation, electronic detection of nanoscale mechanical motion in NEMS was first accomplished by the magnetomotive transduction technique. Here, the detection loop containing the nanoscale structure is placed in a magnetic field. As the structure resonates, the magnetic flux through the detection loop gets modulated due to the motional area change. By picking up the electromotive force (EMF) generated due to the varying flux, one can measure the motion of the NEMS [8]. The electronic background can be greatly reduced by using an on-chip bridge structure [132]. As discussed above in Section 2.2, however, the requirement of high magnetic fields limits the applicability of this technique.

Initial experiments that pushed the limits of electronic displacement sensitivity were motivated by a desire to observe quantum effects on mechanical motion. Researchers engineered displacement detection schemes suitable for cryogenic temperatures. For instance, a DC-biased nanomechanical structure was electrostatically coupled to the island portion of a single-electron transistor (SET), where mechanical motion modulated the impedance of the SET [78,133]. In both these experiments, displacement sensitivities ≤5 $\mathrm{fm}/{\mathrm{Hz}}^{1/2}$ were achieved at low temperatures ≤50 mK. Nanomechanical motion of a miniaturized mechanical device can also be detected by monitoring the current through an atomic point contact or a tunnel junction formed between a sharp tip and the device in question, either on the same chip [134,135] or off board [136,137]. Coupling mechanical devices to superconducting quantum interference devices (SQUID) likewise has enabled extremely precise measurement of mechanical motion, e.g., with a sensitivity of $10\;\mathrm{fm}/{\mathrm{Hz}}^{1/2}$ [138].

Piezoresistive effect—the change of electrical resistance due to mechanical strain—offers possibilities for robust, integrated and room-temperature transducers for motion detection. Piezoresistivity is quantified by the dimensionless gauge factor defined as the ratio of the change in normalized resistance over the strain. Here, one needs to make a distinction between two different mechanisms that generate piezoresistivity: the geometric effect and the resistivity (ρ) change. Deformation of an electrode causes a purely geometric effect: for instance, axial elongation together with the accompanying reduction in cross-section (for a positive Poisson ratio) causes the resistance *R* to increase since R=ρL/A, where ρ is the resistivity, *L* is the length and *A* is the cross-sectional area of the resistor. This geometric effect is relevant for metals which typically have gauge factors between 1 and 2. The other mechanism, the resistivity change, originates from a change in the electronic band configuration of the material due to applied strain. This is usually the dominant effect in piezoresistive semiconductors, in which gauge factors of a few hundred are possible.

**Figure 9:**
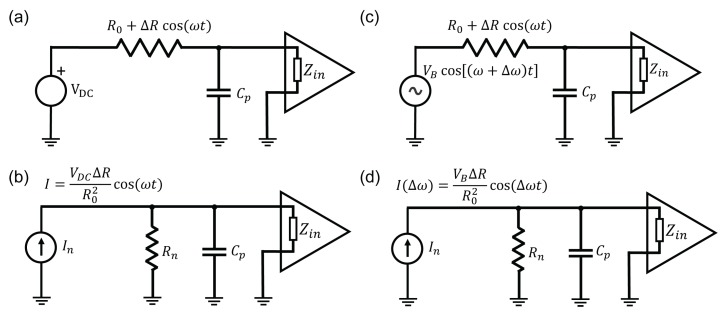
Piezoresistive detection with and without mix-down. (**a**) When a DC bias is used to detect the motion-induced resistance change ΔRcos(ωt), the signal at ω (which is typically at tens of MHz) is effectively short-circuited by the parasitic capacitance in the system (typically pFs); (**b**) alternatively, one may consider the Norton equivalent model for the source: the high frequency current at ω flows through the path of least resistance (the capacitor) to the ground, and signal power transferred to the input impedance of the amplifier is severely limited; (**c**) principle of mix-down detection: instead of a DC voltage, an AC voltage is used to bias the piezoresistance at a frequency ω+Δω, which is slightly different from the mechanical actuation frequency ω. The output signal contains a Δω component (at kHz frequencies), for which the capacitance appears as an open-circuit and a significant portion of the signal appears across the input of the amplifier; (**d**) looking at the Norton equivalent offers further insight: shown here is only the component of current at frequency Δω (there is also the other component at ω which is shorted by the capacitance). Since the current is at kHz frequencies, parasitic capacitances (on the order of pFs) will appear as open-circuit and most of the signal current will flow into the amplifier.

For NEMS applications, both types of the piezoresistive effect have been exploited. Using metallic electrodes, which utilize only the geometric effect, it is easier to obtain resistances close to 50Ω. This is not only important for matching to 50-Ω RF lines, but also for avoiding signal reduction due to parasitic capacitances. Parasitic capacitances in the system (between cables/printed circuit board (PCB) traces/wirebonds carrying the signal and any ground plane nearby) give rise to a low-pass filter with a cut-off frequency at 1/2πRC, where *C* is the total capacitance from the signal path to the ground and *R* is the resistance of the device. Thus, the small electrode (source) resistance *R* is an important advantage of metallic piezoresistive detection. In contrast, silicon based piezoresistive electrodes have resistances of at least a few kΩ and typically a few tens of kΩ. Therefore, it is difficult to directly measure signals from silicon-based piezoresistors due to the RC cut-off (for a 1-kΩ sensor resistance and 1 pF cable capacitance, RC cut-off frequency is 160 kHz). It becomes feasible, however, to obtain an unattenuated output signal by shifting the measurement frequency to smaller values by using a mix-down technique [82,139]. In this important technique, the resistance of the output electrode is biased by an AC voltage, which has a frequency set very close to but slightly different from the mechanical actuation frequency (Figure 9). Mathematically, the resistance of the electrode has both a constant and a dynamical term: $R_{0}+\Delta R\cos\left(\omega t\right)$. When this resistance is biased with a voltage of the form $V_{bias}\left(t\right)=V_{B}\cos\left(\left(\omega +\delta \omega \right)t\right)$, the output current contains a term $I\left(\Delta \omega \right)=\left(V_{B}/R_{0}\right)\left(\Delta R/R_{0}\right)\cos\left(\Delta \omega t\right)$ at the mix-down frequency Δω, which can be set to be a low value (typically 10–100 kHz for a lock-in amplifier based detection). By working at Δω, the RC cut-off due to parasitic capacitances is avoided (Figure 9c,d). Therefore, the mix-down technique allows for piezoresistive detection with semiconductor electrodes, which have gauge factors a few orders-of-magnitude larger and conductances significantly smaller than metallic electrodes.

Piezoresistive detection utilizing the geometric effect at the nanoscale was first demonstrated by Li et al. [140]. In this work, 30-nm thick gold detection electrodes were fabricated on 70-nm thick silicon carbide u-shaped cantilevers. A displacement sensitivity of $39\;\mathrm{fm}/{\mathrm{Hz}}^{1/2}$ was reported. A piezoceramic shaker under the NEMS chip was used to actuate the device. Since low-resistance metallic electrodes were used, mechanical resonances were measured directly with a low-noise amplifier/network analyzer chain without the need for mix-down detection. Metallic piezoresistive detection was also used in tandem with thermo-elastic actuation [75] by placing u-shaped metallic electrodes on both ends of a doubly-clamped beam as shown in Figure 4. While one can optimize the thermo-elastic drive and the piezoresistive detection by fabricating the two electrodes from different materials and with different geometries, it is also possible to use identical electrodes from the same material (e.g., gold) in order to simplify the fabrication process.

An early example of semiconductor-based piezoresistive detection was the work of He et al. [141]. In this work, a bottom-up silicon nanowire was grown between electrically-accessible microtrenches. Although the material exhibits piezoresistivity, it is not straightforward to harness this property for detection, since the total strain over a flexural mode shape is zero to first order (i.e., the top surface extends whereas the bottom surface compresses). However, there is a second order effect: the total elongation of the structure as it vibrates. Since maximal elongation happens twice during one cycle of oscillation, this effect appears at twice the frequency of the nanowire motion (the 2ω term). For this reason, this effect is quadratic with respect to the oscillation amplitude. Using a circuitry capable of actuating the wire at ω (with an off-chip piezoelectric shaker) and detecting the output voltage at 2ω, the authors were able to measure resonances in the very high frequency (VHF) range. To facilitate detection, a mix-down technique was used.

**Figure 10:**
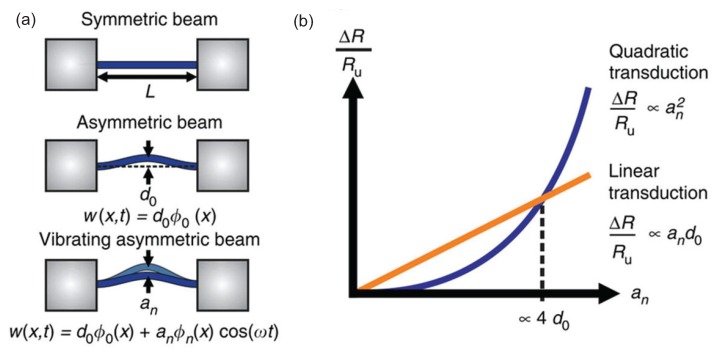
(**a**) Placing an initial static displacement on a beam modifies the motion profile of the beam as it resonates. Mixing between the static and resonant displacement term enables for a readout term at the resonance frequency that is linearly proportional to the initial displacement $d_{0}$ and resonant amplitude $a_{n}$. There is also the quadratic term (proportional to $a_{n}^{2}$ with a frequency 2ω), which emerges from the elongation of the structure; (**b**) linear detection is more sensitive for detecting small amplitude oscillations, specifically as long as the oscillation amplitude is smaller than four times the initial deflection. Reprinted by permission from Macmillan Publishers Ltd.: Nature Communications [142], copyright 2014.

In an interesting variant of this technique, efficient piezoresistive detection through an entire nanowire was accomplished by inducing a static deflection and exciting the structure at resonance [142], as shown in Figure 10. Here, the authors realized that an initial static displacement profile on the structure, $w\left(x\right)=d_{0}\varphi_{0}\left(x\right)$, would generate a linear (at frequency ω) piezoresistive response when a dynamical motion was induced. In this study, the static displacement originated from the fabrication process. When the deflected beam was excited dynamically at the $n^{\mathrm{th}}$ mode, the total displacement profile was given by $w\left(x,t\right)=d_{0}\varphi_{0}\left(x\right)+a_{n}\varphi_{n}\left(x\right)cos\left(\omega t\right)$, where $a_{n}$ is the amplitude and ω is the frequency of the mode with eigenfunction $\varphi_{n}\left(x\right)$. As before, piezoresistive signal is proportional to the strain in the beam, which can be calculated as $\int w^{\prime }\left(x,t\right)dx$. From this expression, two distinct terms arise: a term at 2ω proportional to ${a_{n}}^{2}$—this term is identical to the term measured in [141]. The second term, which is due to the static deflection, occurs at frequency ω and is proportional to both the static deflection $d_{0}$ and $a_{n}$. The ω term dominates the 2ω term as long as $a_{n}>4d_{0}$. Therefore, inducing a large initial deflection on the structure facilitates efficient detection of mechanical motion using piezoresistive detection at the resonance frequency of the structure.

The studies mentioned above [141,142] solved the problem of detecting piezoresistive changes through an entire nanowire. Thus, they were able to extend the technique to very small mechanical structures. There is another option though: to embed semiconductor piezo gauges of desired shapes onto the mechanical structure. One of the first examples of this approach accomplished piezoresistive detection of in-plane motion [80]. Here, degenerately doped silicon structures are fabricated on SOI wafers. Typical device geometry is similar to that shown in Figure 4, where a nearby gate drives the mechanical motion of the entire structure. Piezoresistive transduction occurs at the two small nano-bridges connecting the suspended structure to the side anchors: as the structure moves in plane, one of these bridges experiences compression and the other extension. Consequently, their resistance changes have opposite signs and the mechanical motion can be conveniently read out using a differential measurement. As the source resistances are large, mix down detection is used. This work was also significant because devices were fabricated in a foundry at the wafer scale. This technique was used in several sensing applications, such as single-molecule [29] and single-particle [81] detection, as well as for gas sensing with arrays composed of u-shaped silicon cantilevers [143]. Complementary metal–oxide–semiconductor (CMOS) integration of this device architecture has also been demonstrated [144].

An interesting detection technique, in some ways similar to piezoresistive detection, is employed in carbon nanotube [82] and graphene nanomechanical [6] devices. Here, as the device oscillates back and forth near a gate electrode, the number of charge carriers in the structure gets modulated by electrical gating—which cause the conductance across the device to change. Although this mechanism is different from piezoresistivity, the end result is the same: the dynamic resistance change across the suspended nanostructure enables motion detection. This technique also necessitates the use of mix-down detection since device resistances are in the kΩ range.

Piezoelectric detection was used in combination with piezoelectric actuation (under parametric amplification to avoid cross-talk) [94] as mentioned above in Section 2.2. In a room temperature variant of this technique, piezoelectrically actuated resonators were detected through a capacitively coupled silicon field effect transistor [145] with a displacement sensitivity of $4.4\;\mathrm{pm}/{\mathrm{Hz}}^{1/2}$. Recent experiments with nanoscale resonators with embedded 2-dimensional electron gas (2-DEG) structures also used the piezoelectric effect in order to detect nanomechanical vibrations [146]. To verify the piezoelectric mechanism—which arises due to the underlying crystal structure rather than a mere change in electronic density—the authors fabricated two cantilevers along perpendicular crystal orientations: the readout signals had opposite signs indicating a dependence on the crystal orientation, an anisotropy which signifies piezoelectricity. The experiments were performed at 4.2 K using GaAs/AlGaAs heterostructures and employed electrostatic actuation [147].

The actuation technique based on dielectric gradient force can be reversed to detect the mechanical motion of polarizable resonators [85]. This technique has already been discussed in detail in Section 2.2.

Capacitive detection can also be used in an elaborate way for detecting nanoscale motion of doubly-clamped beam resonators [77]. The main challenge for this technique is that the motional change in capacitance is extremely small, in the atto-Farad range, which is much smaller than parasitic capacitances in the detection circuit. To remedy the situation, the NEMS beam is biased with a DC voltage; when the nanobeam oscillates, its electromechanical impedance can be modeled as an RLC circuit in series with the static coupling capacitance. The total impedance of the system varies significantly between off-resonance and on-resonance states. To read the impedance change due to mechanical motion, one still needs to match the impedance close to the 50Ω impedance of the RF circuit. A simple LC impedance transformation circuit with off-chip components can be used for impedance matching purposes. With this improvement, sufficient electrical contrast is obtained to clearly detect the mechanical resonances in frequency sweeps. Moreover, the technique enables the measurement of many resonators in parallel. Here, each of the many resonators are connected to the external impedance matching circuit through their own coupling capacitances. Individual resonator frequencies are slightly different but close enough such that all resonators can be matched using the same LC tank circuit. In this way, an array of 10 resonators was measured through the same RF line [77]. Capacitive detection with impedance transformation was also used at milliKelvin temperatures for nanoresonator-based read-out of a superconducting charge qubit [148]. Another elaborate way to use capacitive detection for nanoscale motion is to integrate the CMOS readout circuitry monolithically with the NEMS device [149,150,151]. By fabricating the amplifier next to the resonator, the issue of parasitic capacitance is virtually eliminated and an entire measurement system with extremely small device area is obtained. Using this technique, a self oscillating NEMS + CMOS system at 7.8 MHz was demonstrated [150].

As discussed in the optical actuation and detection sections (Section 2.1, Section 3.1 and Section 3.2), coupling between optical cavities and mechanical resonators opens up novel ways to detect as well as dampen (cooling via red-detuning) and drive (amplification via blue-detuning) mechanical motion [42,43,44,152,153,154,155]. These techniques can also be implemented using electromagnetic waves at microwave frequencies rather than at optical frequencies. Instead of optical cavities, microwave resonators in the form of coplanar waveguides or microstriplines can be used. Low-noise microwave generators replace tunable lasers. Since microwave components can be readily integrated and require no geometric alignment, microwave approaches offer practical benefits. There are important drawbacks, however: the momentum of a microwave photon is much smaller than a photon at optical frequencies, hence radiation pressure effect (per photon) is much smaller [156]. Moreover, it is difficult to obtain microwave resonators with high *Q* factors at room temperature due to resistive and dielectric losses. Therefore much of the work in this area has been done at low temperatures using superconducting circuit elements. In typical experiments, a movable electrode (i.e., the nanomechanical resonator) modulates the frequency of the microwave resonator by changing the effective capacitance [157,158,159,160,161,162]. The resulting sidebands of mechanical origin can then be mixed down with the same signal driving the microwave resonator to obtain the mechanical resonance signals. Detailed information on the use of microwave based optomechanics for observing quantum mechanical effects can be found in [156]. In addition to sensitive motion detection, microwave-based cavity mechanics coupled with an optomechanical transducer has allowed for bidirectional conversion between optical and microwave photons, with possible applications in quantum computing and information processing [163,164,165].

Nanomechanical detection has recently been accomplished by using a room-temperature (electrical) microwave resonator. In one of the first examples [166], researchers used a λ/4 microwave resonator, in which input and output ports were coupled inductively through nearby traces on a PCB. A room-temperature *Q* factor of 70 was obtained for the microwave resonator. To couple this microwave resonator to mechanics, a wirebond was used to connect the anti-node region of the microstripline to a gate electrode in close proximity of the dielectric NEMS device. The fringing electric field of the gate electrode samples the space around; the relative permittivity of the NEMS increases the total capacitance of the microwave resonator (Figure 6). With this scheme, a displacement sensitivity of $4.4\;\mathrm{pm}/{\mathrm{Hz}}^{1/2}$ was reported at room-temperature; moreover, cavity induced damping and oscillation were demonstrated. It is also possible to detect both in-plane and out-of-plane motion [87] since the effective capacitance of the microwave resonator changes for both motion directions. Mechanical actuation based on dielectric gradient force can be combined effectively with microwave based detection, by using a bypass capacitor to decouple the GHz range microwave signal from the MHz range mechanical drive signal, as shown in Figure 6d. In a different implementation, the tip of a λ/4 coaxial microwave resonator is exposed, sharpened and placed near a microcantilever [167]. The evanescent field from the resonator probes the motion of cantilever. Mechanical motion modulates the capacitance, produces sidebands in the microwave resonance frequency and is detected by homodyne detection.

An extension of the tunnel-current-based displacement detection is one that relies on the interaction forces between two surfaces in close proximity. Various AFM modalities have been used to detect the motion of micro- and nanomechanical resonators. In these experiments, a resonant mode of the small device under study is excited by an actuator. An AFM cantilever is brought in close proximity of the resonator to probe the oscillations. Both contact mode [7] and non-contact mode AFM [168] have been employed for probing. In some of these experiments, especially in the non-contact mode ones, the strong inherent nonlinearity of the interaction forces between two surfaces in close proximity may offer some advantages from a device perspective [168].

## 4. Conclusions and Outlook

Our focus in this article has been on the development of motion transducers for miniaturized mechanical systems, especially for NEMS devices. It is worth re-emphasizing that MEMS devices owe much of their success to robust motion transducers, such as the interdigitated capacitive motion transducer. While recent progress in the NEMS domain is encouraging, it is hard to imagine a solution, similar to the interdigitated capacitive transducer of MEMS, that might satisfy most of the requirements of future NEMS applications. In this section, we will provide our perspective on the important remaining open problems concerning nanomechanical motion transducers.

One of the key requirements for next generation NEMS transducers is their applicability to NEMS arrays. It is clear that the efficiency obtainable from a single NEMS (e.g., a single NEMS sensor) is rather small, because its effective cross-section (i.e., surface area) is minuscule. The obvious solution is to employ a large number *N* of NEMS distributed over space [37,143], corresponding to an increase in cross-section by a factor ∼*N*. In large arrays, individual device parameters are expected to show large dispersion because of statistical fluctuations in batch fabrication. (This can clearly be seen in the measurements in Figure 1, where nominally identical nanomechanical beams display different fundamental resonance frequencies.) Thus, operating arrays and processing data from arrays with nonuniform parameters quickly becomes challenging as *N* grows.

A second requirement is the operability of the transducer in a liquid buffer. Some foreseeable bio-sensing applications of NEMS are in liquid buffers. In a liquid, the viscous dissipation changes the dynamics of the NEMS and makes available signals significantly smaller than those in air (or vacuum) due to a reduction in the *Q* factor, as discussed in Section 1.2. Furthermore, electrical actuation and detection approaches may not work well due to the presence of ions in buffers.

Ultimately, one needs to pursue the highest available displacement detection sensitivities. Even though shot noise-limited displacement sensitivity has been demonstrated in the optical domain, this fundamental limit has been achieved under the most optimal conditions. It would be beneficial to have a transduction mechanism and develop transducers that can attain such high sensitivities routinely. Especially for microwave resonators, in which high mechanical stiffnesses result in extremely small motions, novel approaches are needed for probing nanomechanical motion with high temporal and spatial resolution.

## Abbreviations

The following abbreviations are used in this manuscript:

AC	Alternating current
AFM	Atomic force microscopy
CMOS	Complementary metal–oxide–semiconductor
DC	Direct current
EMF	Electromotive force
MBE	Molecular beam epitaxy
MEMS	Micro-electro-mechanical systems
NEMS	Nano-electro-mechanical systems
NOEMS	Nano-opto-electro-mechanical systems
NOMS	Nano-opto-mechanical systems
PCB	Printed circuit board
*Q* or *Q* factor	quality factor
RF	Radiofrequency
SEM	Scanning electron microscopy
SET	Single-electron transistor
SOI	Silicon-on-insulator
SPM	Scanning probe microscopy
SQUID	Superconducting quantum interference devices
VHF	Very high frequency
WGM	Whispering gallery mode
2-DEG	2-Dimensional electron gas
